# Induction and maintenance of DNA methylation in plant promoter sequences by apple latent spherical virus-induced transcriptional gene silencing

**DOI:** 10.3389/fmicb.2014.00595

**Published:** 2014-11-10

**Authors:** Tatsuya Kon, Nobuyuki Yoshikawa

**Affiliations:** Plant Pathology Laboratory, Faculty of Agriculture, Iwate UniversityMorioka, Japan

**Keywords:** apple latent spherical virus, virus vector, agroinoculation, TGS, DNA methylation, epigenetic modification

## Abstract

Apple latent spherical virus (ALSV) is an efficient virus-induced gene silencing vector in functional genomics analyses of a broad range of plant species. Here, an *Agrobacterium*-mediated inoculation (agroinoculation) system was developed for the ALSV vector, and virus-induced transcriptional gene silencing (VITGS) is described in plants infected with the ALSV vector. The cDNAs of ALSV RNA1 and RNA2 were inserted between the cauliflower mosaic virus 35S promoter and the NOS-T sequences in a binary vector pCAMBIA1300 to produce pCALSR1 and pCALSR2-XSB or pCALSR2-XSB/MN. When these vector constructs were agroinoculated into *Nicotiana benthamiana* plants with a construct expressing a viral silencing suppressor, the infection efficiency of the vectors was 100%. A recombinant ALSV vector carrying part of the 35S promoter sequence induced transcriptional gene silencing of the green fluorescent protein gene in a line of *N. benthamiana* plants, resulting in the disappearance of green fluorescence of infected plants. Bisulfite sequencing showed that cytosine residues at CG and CHG sites of the 35S promoter sequence were highly methylated in the silenced generation zero plants infected with the ALSV carrying the promoter sequence as well as in progeny. The ALSV-mediated VITGS state was inherited by progeny for multiple generations. In addition, induction of VITGS of an endogenous gene (*chalcone synthase*-*A*) was demonstrated in petunia plants infected with an ALSV vector carrying the native promoter sequence. These results suggest that ALSV-based vectors can be applied to study DNA methylation in plant genomes, and provide a useful tool for plant breeding via epigenetic modification.

## INTRODUCTION

In plants, small RNAs play an important role in the RNA silencing pathway, which interferes with gene expression and also acts against viral infection (Baulcombe, 2004; Kon and Ikegami, 2009). Small RNAs also have an epigenetic mechanism that results in transcriptional gene silencing (TGS) of an endogenous gene through DNA methylation (Law et al., 2010). DNA cytosine methylation is an important epigenetic marker for TGS and controls development and gene expression, and also acts in genome defense against molecular parasites (e.g., viruses, transposons; Liu et al., 2009; Zemach et al., 2010). In plants, the cytosine at CG, CHG, and CHH sites (where H is A, C, or T) can be methylated by DNA methyltransferases (Cokus et al., 2008; Law and Jacobsen, 2010). RNA-directed DNA methylation (RdDM), an epigenetic process in plants, requires a trigger for the production of RNAs, including transcripts of endogenous RNA-dependent RNA polymerases and viral/viroid replication intermediates. In an initial step of RdDM, aberrant RNAs are converted into double-stranded RNA (dsRNA), and then this dsRNA is processed by a Dicer-like protein to generate small RNAs (Pontes et al., 2006; Wierzbicki et al., 2008). Small RNAs are loaded onto Argonaute (AGO) proteins and an AGO-RNA complex directs *de novo* DNA methylation at the target DNA loci (Wierzbicki et al., 2009; Gao et al., 2010). In *Arabidopsis*, two maintenance classes of DNA methyltransferases [*METHYLTRANSFERASE 1* (*MET1*) and *CHROMOMETHYLASE 3* (*CMT3*)] catalyze cytosine methylation at a CG site and at CHG/CHH sites, respectively, whereas *DOMAINS REARRANGED METHYLTRANSFERASE 2* (*DRM2*) is a member of the *de novo* class of DNA methyltransferases, and is required both for maintenance and initiation of DNA methylation (Cao et al., 2003; Chan et al., 2005). Thus, RdDM induces epigenetic modifications of homologous sequences, and in the gene promoter region, suppresses gene expression at the transcriptional level. Virus-induced transcriptional gene silencing (VITGS) is one of the newer methods for induction of epigenetic modifications in plants (Kanazawa et al., 2011). In plants, DNA methylation can be induced by infection with viruses or viroids. VITGS suppresses gene expression at the level of transcription in the nucleus; moreover, targeting non-coding regions (e.g., gene promoters) results in methylation that is inherited, whereas targeting coding regions also results in methylation, but is not inherited (Jones et al., 2001).

Apple latent spherical virus (ALSV) is a member of the genus *Cheravirus* in the family *Secoviridae*. It has a bipartite single-stranded genome (RNA1 and RNA2) encapsidated into three capsid proteins (Vp25, Vp20, and Vp24; Li et al., 2000; Le Gall et al., 2007). ALSV infects a broad range of plant species and does not induce any obvious symptoms in most host plants (Igarashi et al., 2009). ALSV vector can effectively induce systemic virus-induced gene silencing (VIGS) because ALSV spreads extensively into growing regions of infected plants (Igarashi et al., 2009; Yamagishi et al., 2011). VIGS down-regulates gene expression in a sequence-specific manner following infection with viral vectors carrying fragments of plant genes (Senthil-Kumar and Mysore, 2011). VIGS can rapidly allow identification of a loss-of-function phenotype. Therefore, a modified ALSV vector is used for analysis of VIGS of endogenous genes in plants (Yaegashi et al., 2007; Igarashi et al., 2009; Yamagishi and Yoshikawa, 2009). Construction of the ALSV RNA2 vector, containing the cauliflower mosaic virus (CaMV) 35S RNA promoter and nopaline synthase terminator (NOS-T) sequence cloned into a pUC-based plasmid, was previously described (Li et al., 2004). Although these viral cDNAs were infectious, the infectivity is not high enough for direct inoculation to many plant species. Cloned ALSV cDNAs first need to be inoculated into *Chenopodium quinoa* plants by mechanical inoculation, and then the virus propagated in *C. quinoa* plants is used as an inoculum for target plants. Agroinoculation methods have been established to introduce many plant viruses into plant tissues because *Rhizobium radiobacter* (previously *Agrobacterium tumefaciens*) can easily introduce viral genomes and foreign genes into plants by means of a simple inoculation method (Vaghchhipawala et al., 2011).

In this study, two ALSV RNA2 vectors were first cloned into a binary plasmid vector, i.e., one that allows a target sequence to be inserted between multiple cloning sites in the coding region of ALSV RNA2, as previously reported (Li et al., 2004), and other modified vectors that allow a target sequence to be inserted in the 3’-non-coding region of ALSV RNA2. Then we developed an agroinoculation system of ALSV-based vectors that are highly infectious in experimental host *Nicotiana benthamiana* plants. We also report that an ALSV vector carrying part of the 35S promoter sequence was able to induce TGS of the green fluorescent protein (GFP) gene in a transgenic line of *N. benthamiana* plants, and the ALSV-mediated VITGS state was inherited by progeny for multiple generations. Induction of VITGS of an endogenous gene (*chalcone synthase*-*A*) was also demonstrated in petunia plants infected with an ALSV vector carrying the native promoter sequence.

## MATERIALS AND METHODS

### CONSTRUCTION OF ALSV-BASED VECTORS FOR VIGS/VITGS

Apple latent spherical virus vectors (pEALSR1 and pEALSR2), previously constructed using the CaMV 35S promoter and the NOS-T in the modified pE18PGT plasmid (Li et al., 2004), were used in this study. The ALSV RNA cDNAs containing the CaMV 35S promoter and the NOS-T sequences were cloned in the binary vector pCAMBIA1300 (Hajdukiewicz et al., 1994) for transformation as described below.

**FIGURE 1:**
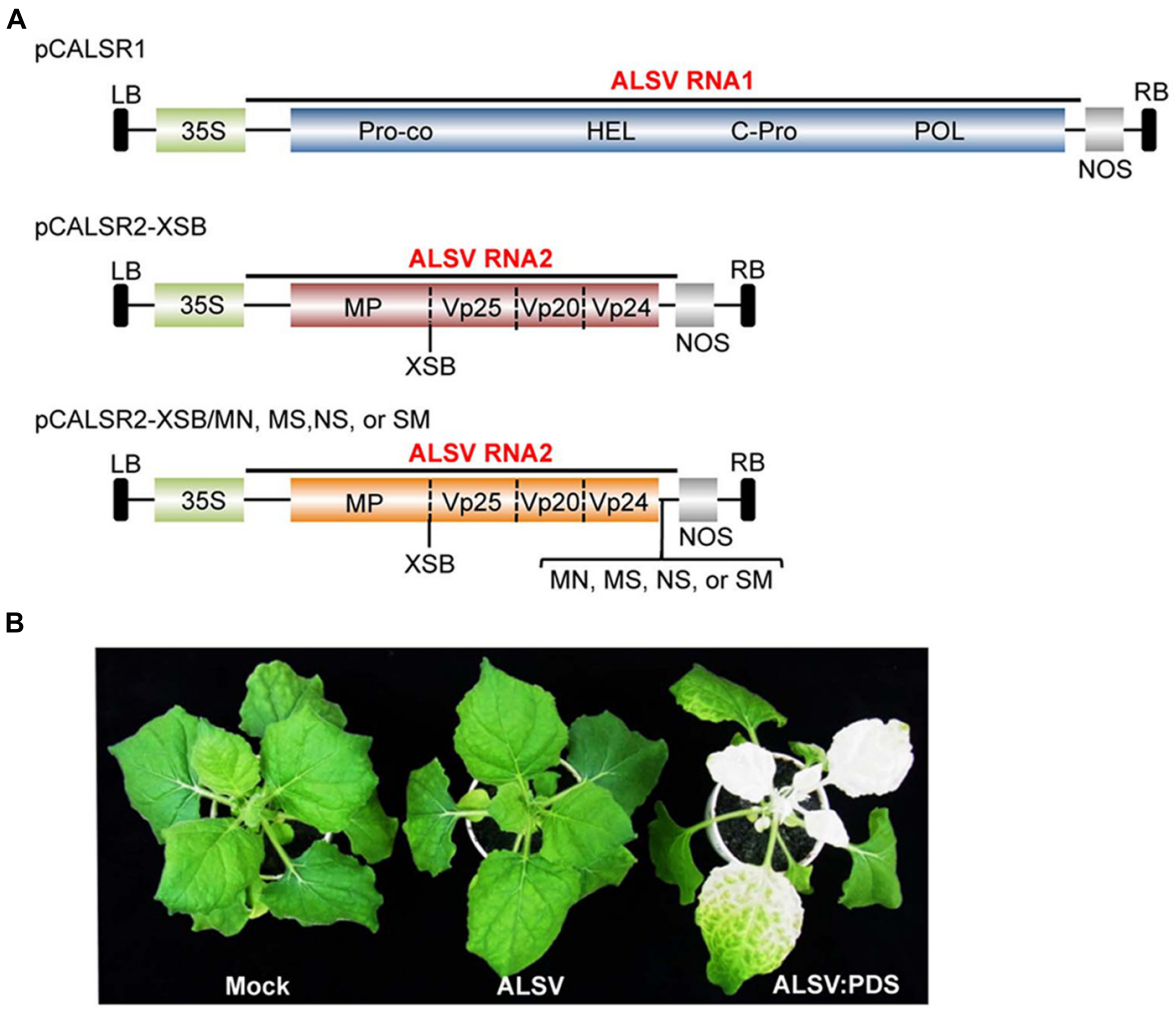
**Schematic representation of infectious clones of ALSV RNAs and infectivity of ALSV-based vector in *N. benthamiana* plants. (A)** The infectious ALSV RNA1 and RNA2 cDNA sequences (Li et al., 2004) were respectively, introduced between the CaMV 35S RNA promoter (35S) and the nopaline synthase terminator (NOS) within the left and right borders (LB and RB) of the pCAMBIA1300 binary vector to produce pCALSR1 and pCALSR2-XSB. Restriction sites (*Mlu* I-*Nco* I [MN], *Mlu* I-*Sal* I [MS], *Nco-Sal* I [NS], and *Sal* I-*Mlu* I [SM]) were respectively, introduced into pALSR2-XSB immediately downstream of the translation stop codon of the 108K open reading frame to generate pCALSR2-XSB/MN, pCALSR2-XSB/MS, pCALSR2-XSB/NS, and pCALSR2-XSB/SM. The target genes can be cloned into the *Xho* I, *Sma* I, and *Bam* HI (XSB) restriction sites between the MP and Vp25 duplicate cleavage sites or into the MN, MS, NS, or SM restriction sites immediately downstream of the translation stop codon of the the 108K open reading frame. The open reading frames of ALSV represent the protease co-factor (Pro-co), NTP-binding helicase (HEL), cysteine protease (C-Pro), RNA polymerase (POL), movement protein (MP), and three capsid proteins (Vp25, Vp20, and Vp24). **(B)** *N. benthamiana* plants were inoculated with empty vector (Mock), pCALSR1 + pCALSR2-XSB (ALSV), and pCALSR1 + pCALSR2-XSB:NbPDS (ALSV:PDS) by agroinoculation. Infection with ALSV (pCALSR1 + pCALSR2-XSB) is symptomless and infection with ALSV:PDS (pCALSR1 + pCALSR2-XSB:NbPDS) causes photobleaching of leaves by 14 days post-inoculation.

First, the *hygromycin phosphotransferase* (*HPT*) gene was removed from pCAMBIA1300 by digestion with *Xho* I, and then blunt-ended with T4 DNA polymerase. For constructing the ALSV RNA1 vector, an ∼0.5 kb *Bam* HI-*Eco* RI fragment containing ALSV RNA1 3′-cDNA including poly-A sequences and the NOS-T from pEALSR1 was cloned into pCAMBIA1300, from which was removed the *HPT* gene to generate pCAR1-3-NT. A second fragment released from pEALSR1, an ∼1.9 kb *Hin*d III-*Xba* I fragment containing the CaMV 35S promoter and the ALSV RNA1 5′-cDNA, was cloned into pCAR1-3-NT to generate pCCaMAR1-5/3-NT. Finally, the third fragment released from pEALSR1, an ∼6.2 kb *Sac* I-*Bam* HI fragment containing ALSV RNA1 cDNA, was cloned into modified pCCaMAR1-5/3-NT to generate pCALSR1 (**Figure 1A**).

For constructing the ALSV RNA2 vector, an ∼0.9 kb *Kpn* I-*Eco* RI fragment containing ALSV RNA2 3′-cDNA including poly-A sequences and the NOS-T from pEALSR2 was cloned into pCAMBIA1300 as described above to generate pCAR2-3-NT. A second ∼2.2 kb *Hin*d III-*Bam* HI fragment containing the CaMV 35S promoter and the ALSV RNA2 5′-cDNA was cloned into pCAR2-3-NT to generate pCCaMAR2-5/3-NT. Finally, the third ∼1.8 kb *Bam* HI-*Spe* I fragment containing ALSV RNA2 cDNA was cloned into modified pCCaMAR2-5/3-NT to generate pCALSR2-XSB (**Figure 1A**).

For constructing the modified ALSV RNA2 vector, four restriction enzyme sites, *Mlu* I-*Nco* I, *Mlu* I-*Sal* I, *Nco* I-*Sal* I, or *Sal* I-*Mlu* I, were introduced into pCALSR2-XSB immediately downstream of the translation stop codon of the 108 K open reading frame using artificial gene synthesis technology to generate pCALSR2-XSB/MN, pCALSR2-XSB/MS, pCALSR2-XSB/NS, and pCALSR2-XSB/SM (**Figure 1A**).

To construct the ALSV RNA2 vector carrying the *phytoene desaturase* (*PDS*) gene of *N. benthamiana*, the sequence of the *PDS* gene was amplified by RT-PCR using the specific P1/P2 primer pair (**Table 1**). The PCR product was cloned and sequenced. The cloned DNA was digested with *Xho* I and *Bam* HI, and then the digested *PDS* cDNA fragment was cloned into pCALSR2-XSB, which had been digested with *Xho* I and *Bam* HI, to produce pCALSR2-XSB:NbPDS. To construct the ALSV RNA2 vector carrying the petunia *CHS-A* gene, the coding sequence of the *CHS-A* gene (Morita et al., 2012) was amplified by RT-PCR using the specific P3/P4 primer pair (**Table 1**). The PCR product was cloned and sequenced. The cloned DNA was digested with *Sal* I and *Bam* HI, and then the digested *CHS-A* cDNA fragment was cloned into pCALSR2-XSB, which had been digested with *Xho* I and *Bam* HI, to produce pCALSR2-XSB:CHS-A.

**Table 1:** Primer pairs used for PCR.

Oligonucleotide (forward/reverse)	Sequence (5′–3′)	
	Forward	Reverse
P1/P2	CTCGAGCTTTCGATGCAGTGC	GGATCCCTCTTTCCAGTCTTCAGGC
P3/P4	CCCGTCGACATGCCTGGGTGTGACTATC	CCCGGATCCCAAAGGCCTCTCGACTCC
P5/P6	ACGCGTGAGGAAGGGTCTTGCGAAGG	CCATGGAGACTTTTCAACAAAGGG
P7/P8	CTCGAGATGGAATTGAACGTAGGTGC	GGATCCGAAAGCACCTTCCGCCCATTC
P9/P10	CTCGAGATGGAACGAGCTATACAAGG	GAGCTCTTACTCGCTTTCTTTTTCG
P11/P12	CCCGTCGACCTGATGCTAGAAGTGACAG	CCCACGCGTGGTAGATAGTAATCACGTG
P13/P14	TGAGAGTTCTCTAAATAAGGGAGGC	CAGCTTTGAGCAATCTGAACCTGGC
P15/P16	CACAATGATAGGAAGAGCCGAC	CAAGGGAACGGGCTTGGCAGAATC
P17/P18	GGGAACTACAAGACACGTGC	AAGGGCAGATTGTGTGGAC
P19/P20	GAGAAGAGCCTTGAGGAAGC	GTTCCTAAACCTTCTTTGGC
P21/P22	AAGTAATAGAGATTGGAGT	CCCATTAACATCACCATCTAATTCAAC
P23/P24	GAAAATCTTCGTCAACATGGTGGAG	CAACTCCAGTGAAAAGTTCTTCTCC
P25/P26	TAATGAGATGAGTTTATATAG	CACATGCGCTTAAATTTCTCC
P27/P28	AAAAGGATACTACAATTTGG	CACGTTGTGCCTTACGATACTCCTC

### CLONING OF RNA SILENCING SUPPRESSORS

The P19 gene of tomato bushy stunt virus (accession number M21958) was synthesized by artificial gene synthesis (FASMAC Co. Ltd., Atsugi, Japan), and then digested with *Xba* I and *Sac* I and cloned into the pBIN3 binary vector (kindly provided by Professor Masato Ikegami) to produce pBIN3:P19. Binary plasmids expressing silencing suppressors 2b of cucumber mosaic virus (CMV)-pepo (pBE2113:2b; Yaegashi et al., 2007), AC2 of tomato mosaic Havana virus (pBIN3:AC2, kindly provided by Professor Robert Gilbertson), 50 KP of apple chlorotic leaf spot virus (pBE2113:50K; Yaegashi et al., 2007), and HC-Pro of potato virus Y (pBIN61:HC-Pro, kindly provided by Professor David Baulcombe) were used.

### AGROINOCULATION

For agroinoculation, pCALSR1- and pCALSR2-based vectors were transformed into *R. radiobacter* strains GV3101::pMP90 and C58C1, respectively. The binary plasmids pBIN3, pBIN61:HC-Pro, pBE2113:2b, pBIN3:P19, pBE2113:50K, and pBIN3:AC2 were transformed into *R. radiobacter* strain C58C1 (pCH32).

Agroinoculation was carried out as described by Kon et al. (2009) with slight modifications. *R. radiobacter* strains were grown in LB medium containing the appropriate antibiotics at 30°C. Cells were pelleted and resuspended in agroinduction buffer (10 mM MES, pH 5.7, 10 mM MgCl_2_, and 200 μM acetosyringone) to a final OD 600 = 1.0. The cell suspension was incubated for 3 h and then infiltrated onto the abaxial leaf surface of *N. benthamiana* and petunia (3–5 leaf stages) using a 1 ml syringe without a needle.

### INDUCTION OF TGS

The CaMV 35S promoter (nt –343 to –32) was amplified by PCR with primers P5/P6 (**Table 1**), and then cloned into the pGEM-T Easy vector and sequenced. Then the CaMV 35S promoter (nt –343 to –32) was cloned into *Mlu* I/*Nco* I-digested pCALSR2-XSB/MN to generate pCALSR2-XSB/MN:35S. The *2b* and *P19* genes were respectively, amplified from pBE2113:2b and pBIN3:P19 using the primer pairs P7/P8 and P9/P10 (**Table 1**). PCR products were cloned into the pGEM-T Easy vector (Promega) and sequenced to confirm the absence of sequencing errors in the cloned genes in the constructs. Each suppressor gene was cloned between the *Xho* I and *Bam* HI sites of pCALSR2-XSB/MN:35S.

Total genomic DNA was extracted from petunia cv. Red Star, and the *CHS-A1* promoter of Red Star petunia (nt –447 to –52, Morita et al., 2012) was amplified by PCR with primers P11/P12 (**Table 1**). The amplified PCR product of *CHS-A1* promoter gene was cloned into the pGEM-T Easy vector and sequenced. A fragment of the *CHS-A1* promoter construct was cloned into *Mlu* I/*Nco* I-digested pCALSR2-XSB/MN to produce pCALSR2-XSB/MN:CHS-Apro. The cloned DNA was transformed into *R. radiobacter* strain C58C1 and *N. benthamiana* line 16c plants (kindly provided by Professor David Baulcombe) or agroinoculated into petunia plants as described above.

### RNA ANALYSIS

Total RNA was extracted from agroinoculated plants using an RNeasy Plant Mini Kit (Qiagen) and then treated with DNase I. For RT-PCR, first-strand cDNA was synthesized by M-MuLV reverse transcriptase (New England Biolabs) with random 6-mers. RT-PCR was carried out with ALSV RNA2 specific primers (P13/P14), 26S rRNA specific primers (P15/P16), *GFP* specific primers (P17/P18), or *CHS-A* specific primers (P19/P20; **Table 1**). Isolation of low molecular weight RNA and detection of siRNAs by northern blotting were as described by Yaegashi et al. (2007). The membranes were hybridized with digoxigenin (DIG)-labeled RNA probes for the CaMV 35S promoter sequence (nt –343 to –32) and the *CHS-A1* promoter gene (nt –447 to –52) of petunia cv. Red Star. siRNAs were immunodetected with anti-DIG Fab fragments coupled to alkaline phosphatase using an ImageQuant LAS4000 imager (GE Healthcare).

### BISULFITE SEQUENCING AND DNA METHYLATION ANALYSIS

Total genomic DNA was extracted from leaves using a DNeasy plant mini kit (Qiagen). For bisulfite sequencing, 1 μg of genomic DNA was treated with bisulfite using an EpiTect bisulfite kit (Qiagen) with slight modifications. The bisulfite-treated DNA was purified, and then the targeted regions were PCR-amplified. All primers for bisulfite sequencing analysis are listed in **Table 1**. For the CaMV 35S promoter in *N. benthamiana* line 16c plants, the target DNA region was amplified by a first round of PCR with primers P21/P22. The product from the first reaction was used for a second round of PCR with primers P23/P24. For the *CHS-A* promoter in petunia plants, the target DNA regions, *CHS-A1* and *CHS-A2*, were amplified by a first round of PCR with primers P25/P26. The product from the first reaction was used for a second round of PCR with primers P27/P28. To determine whether the cytosine from the unmethylated DNA was converted completely to uracil by bisulfite treatment, unmethylated plasmid DNA containing the CaMV 35S promoter sequence was mixed with total plant genomic DNA, and then analyzed using bisulfite sequencing. The unmethylated plasmid DNA was amplified by PCR after treating with bisulfite and the cloned DNA showed conversion of cytosines to uracils (thymidines). The PCR products were cloned into the pGEM-T Easy vector and at least 10 clones per each position were sequenced. The sequences were aligned and analyzed by Kismeth software (Gruntman et al., 2008).

## RESULTS

### CONSTRUCTION OF ALSV-BASED VECTORS IN A BINARY PLASMID AND AGROINOCULATION

Li et al. (2004) reported the construction of ALSV RNA1 and RNA2 vectors in a pUC-based expression cassette vector (pEALSR1 and pEALSR2L5R5). In this study, the cDNAs of ALSV RNA1 in pEALSR1 and ALSV RNA2 in pEALSR2L5R5 were inserted into binary vector pCAMBIA1300, resulting in pCALSR1 and pCALSR2-XSB, respectively (**Figure 1A**). When pCALSR1 and pCALSR2-XSB were agroinoculated together into *N. benthamiana* plants, they had a 100% infection rate (**Table 2**). Infected *N. benthamiana* plants did not show any obvious symptoms (**Figure 1B**; ALSV), as described before (Yamagishi et al., 2011).

**Table 2:** Effects of viral silencing suppressors on infectivity of apple latent spherical virus vectors following agroinoculation of *Nicotiana benthamiana*.

Inoculum	Viral suppressor^a^	Infectivity^b^
pCALSR1 + pCALSR2-XSB	–	10/10
pCALSR1 + pCALSR2-XSB/MN	–	2/10
pCALSR1 + pCALSR2-XSB/MS	–	2/10
pCALSR1 + pCALSR2-XSB/NS	–	2/10
pCALSR1 + pCALSR2-XSB/SM	–	2/10
pCALSR1 + pCALSR2-XSB/MN + pBE2113:2b	2b	10/10
pCALSR1 + pCALSR2-XSB/NS + pBIN3:AC2	AC2	10/10
pCALSR1 + pCALSR2-XSB/MS + pBIN3:P19	P19	10/10
pCALSR1 + pCALSR2-XSB/SM + pBE2113:50K	50KP	10/10
pCALSR1 + pCALSR2-XSB/SM + pBIN61:HC-Pro	HC-Pro	10/10

*^a^RNA silencing suppressors expressed from co-infiltrated plasmid DNAs. The symbols 2b, AC2, P19, 50KP, and HC-Pro are suppressor proteins of CMV, ToMHV, TBSV, ACLSV, and PVY, respectively.*

*^b^Number of infected plants/inoculated plants based on the detection of viral RNA by RT-PCR. Sum of two independent experiments.*

Apple latent spherical virus-RNA vectors, which have cloning sites in the 3′ non-coding region of RNA2, were developed in this study (**Figure 1A**). When ALSV RNA-based vectors having different restriction enzyme sites (pCALSR2-XSB/MN, pCALSR2-XSB/MS, pCALSR2-XSB/NS, and pCALSR2-XSB/SM) were co-agroinoculated with pCALSR1 into *N. benthamiana*, 20% of plants were infected with each ALSV RNA2 construct (**Table 2**). ALSV Vp24 protein is a weak suppressor of local RNA silencing but a potential suppressor of systemic RNA silencing in plants (Yaegashi et al., 2007). The modified ALSV RNA2-based vectors may cause reduced accumulation of viral RNA during the initial infection stage, and be affected by RNA silencing in plants inoculated with them. To improve the efficiency of infection by agroinoculation of the ALSV-based vector system, RNA silencing suppressors were co-expressed in *N. benthamiana* plants. When pCALSR1 and pCALSR2-XSB/MN were co-agroinoculated with a DNA construct transiently expressing a viral RNA silencing suppressor (pBE2113:2b, pBIN3:AC2, pBIN3:P19, pBE2113:50K, or pBIN61:HC-Pro) into *N. benthamiana* plants, all the inoculated plants were infected, indicating that co-expression with RNA silencing suppressors can increase the infection efficiency of pCALSR2-XSB/MN by agroinoculation from 20 to 100% (**Table 2**). The presence of viral RNAs was detected by RT-PCR using specific primers, and when upper leaves from plants systemically infected with virus were homogenized, subsequent sap-inoculation led to successful infections. When pCALSR2-XSB:NbPDS containing a *PDS* gene fragment of *N. benthamiana* was inoculated with pCALSR1, photobleaching of upper leaves appeared 14 days after inoculation (**Figure 1B**; ALSV:PDS), indicating that virus derived from ALSV RNA1 and modified RNA2-based vectors had systemically infected the plant. These results showed that most plant RNA silencing suppressors increase ALSV vector infectivity. Furthermore, agroinoculated *N. benthamiana* plants provided a source of viral inoculum for subsequent inoculation of other experimental host plants.

### INDUCTION OF VITGS OF THE CAMV 35S TRANSGENE PROMOTER BY THE ALSV VECTOR

**FIGURE 2:**
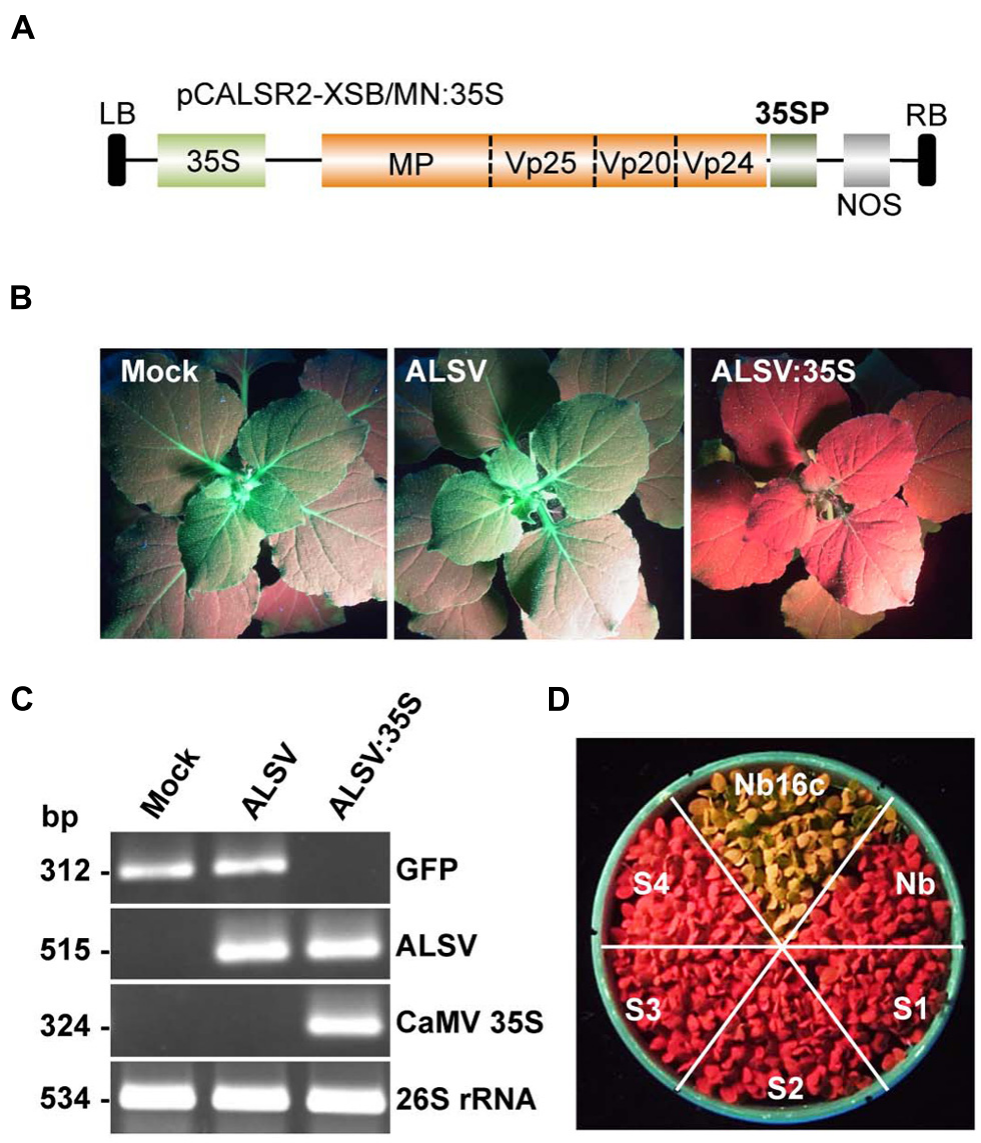
**Induction of TGS by the ALSV vector and its inheritance. (A)** Schematic representation of ALSV vector containing part of the 35S promoter (35SP; pCALSR2-XSB/MN:35S). **(B)** *N. benthamiana* line 16c plants expressing GFP and mock-agroinoculated (Mock), or agroinoculated with pCALSR1 + pCALSR2-XSB (ALSV) or pCALSR1 + pCALSR2-XSB/MN:35S (ALSV:35S). Plants were photographed under UV illumination with a yellow filter. The red fluorescence reveals that TGS of GFP occurred in ALSV:35S-infected tissues. **(C)** Analysis of mRNAs in agroinoculated *N. benthamiana* line 16c plants by RT-PCR. Total plant RNA was extracted from plants agroinoculated with empty vector (lane 1), ALSV (lane 2), or ALSV:35S (lane 3). **(D)** The silenced generations 1–4 (S1–S4) seedlings derived from ALSV:35S infected plants (S0), showing disappearance of GFP fluorescence. The progeny of non-transgenic *N. benthamiana* plants (Nb) and mock-inoculated *N. benthamiana* line 16c (Nb16c) are shown as controls.

To investigate whether the ALSV vector induces DNA methylation, DNA methylation patterns in the CaMV 35S promoter region of transgenic *N. benthamiana* line 16c plants expressing GFP were analyzed. The CaMV 35S promoter (nt –343 to –32) was cloned into the ALSV RNA2-based vector (pCALSR2-XSB/MN) to produce pCALSR2-XSB/MN:35S (**Figure 2A**), and then recombinant ALSV (ALSV:35S) was inoculated into line 16c plants expressing GFP under the control of the CaMV 35S promoter. When a wild-type ALSV and ALSV:35S were inoculated into line 16c plants, mock- or ALSV-inoculated line 16c plants maintained green fluorescence whereas line 16c plants inoculated with ALSV:35S showed a loss of green fluorescence by 3 weeks post-inoculation (**Figure 2B**). RT-PCR failed to detect any GFP mRNA from line 16c plants inoculated with ALSV:35S (**Figure 2C**). These results suggest that the reduction in GFP mRNA is the result of VITGS due to ALSV:35S infection.

Seeds from line 16c plants showing a loss of GFP fluorescence following inoculation with ALSV:35S were harvested to investigate whether VITGS induced by the ALSV vector is inheritable by analyzing the progeny [named silenced generation 1 (S1) plants] from ALSV:35S-infected plants [silenced generation 0 (S0) plants]. None of the S1 progeny obtained from S0 plants showed GFP fluorescence, as shown by the red color in **Figure 2D**. The progeny of the S2–S4 generations, which were free from ALSV infection, also did not show GFP fluorescence (**Figure 2D**). These results indicate that the ALSV vector carrying a plant promoter gene induces DNA methylation of genomic DNA and transcriptionally suppresses the target gene, and that VITGS can be inherited by the next generation.

**FIGURE 3:**
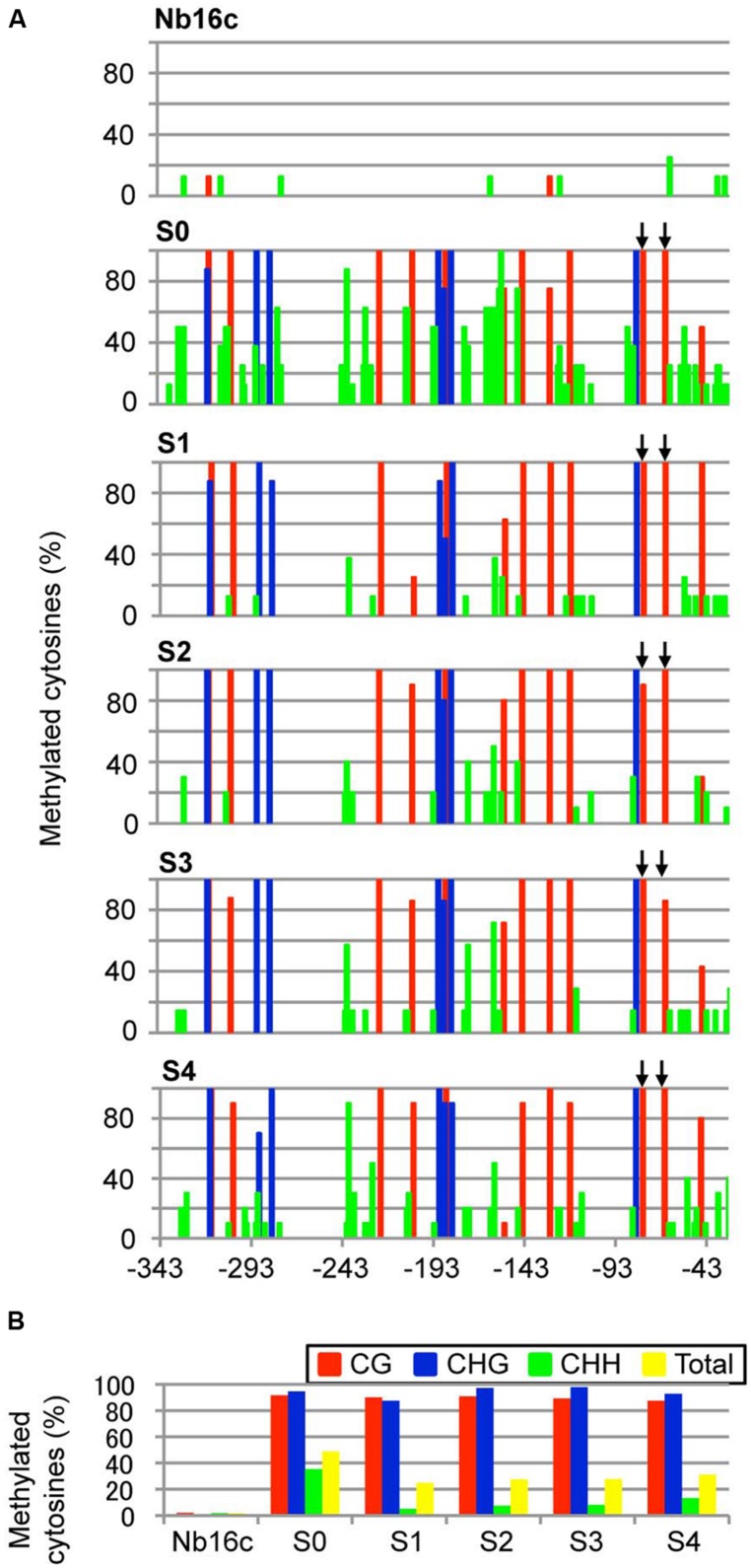
**Cytosine methylation status of the CaMV 35S promoter region. (A)** Total plant DNA from mock-agroinoculated *N. benthamiana* line 16c plants (Nb16c), Nb16c plants infected with ALSV:35S (S0), and progeny from four generations of ALSV:35S-infected Nb16c plants (S1, S2, S3, and S4) were treated with bisulfite. The arrows indicate elements within the domain of activation sequences. **(B)** Summary of bisulfite sequencing analysis. The red, blue, green, and yellow bars respectively represent the percentage of cytosines methylated at the CG, CHG, CHH, and all contexts.

Next, the DNA methylation patterns of the CaMV 35S promoter in ALSV:35S-infected line 16c plants were analyzed by bisulfite sequencing. The target region (nt –343 to –32) of the CaMV 35S promoter contains 12 CG, 7 CHG, and 62 CHH sites. The genomic DNA was treated with bisulfite. In mock-inoculated plants, the cytosine residue at the CHG site was never methylated and only a small number of cytosine residues at the CG and CHH sites were methylated (2.1 and 1.8%, respectively; **Figures 3A,B**). In contrast, in plants agroinoculated with ALSV:35S, the CG and CHG sites were highly methylated (91.7 and 95.6%, respectively). The cytosine residue of the CHH site was also methylated in 35.3% of plants (**Figures 3A,B**). In S1 progeny from ALSV:35S-infected line 16c plants (S0 plants), cytosine methylation patterns were highly maintained at the CG and CHG sites, in 90.6 and 87.5% of plants, respectively (**Figures 3A,B**). On the other hand, cytosine methylation at CHH sites of S1 plants was 5.1%, low compared with S0 plants (35.3%; **Figures 3A,B**).

The region of the CaMV 35S promoter (nt –343 to –32, corresponding to defining the transcriptional start site as zero) has a transcriptional regulation domain, which contains two tandem repeats (TGACG, nt –82 to –78 and –70 to –66), named the activation sequence 1 element (Benfey and Chua, 1990). The activation sequence factor binds to the activation sequence 1 element (Kanazawa et al., 2007). Bisulfite sequencing revealed that cytosine methylation occurred 100% at the CG site in the activation sequence 1 element of the CaMV 35S promoter both in ALSV:35S-infected S0 plants and in S1 plants (**Figures 3A,B**). The DNA methylation patterns of the CaMV 35S promoter in the progeny (S2–S4) were analyzed by bisulfite sequencing. All plants of the S1–S4 generations showed cytosine methylation patterns that were highly maintained at the CG (>89%) and CHG (>94%) sites (**Figures 3A,B**). Thus, an ALSV-based VITGS vector could induce both DNA methylation and TGS in the homologous promoter region, suggesting that cytosine methylation at CG and CHG sites is highly inherited by subsequent generations.

### RNA SILENCING SUPPRESSOR INHIBITS ALSV-MEDIATED VITGS IN PLANTS

**FIGURE 4:**
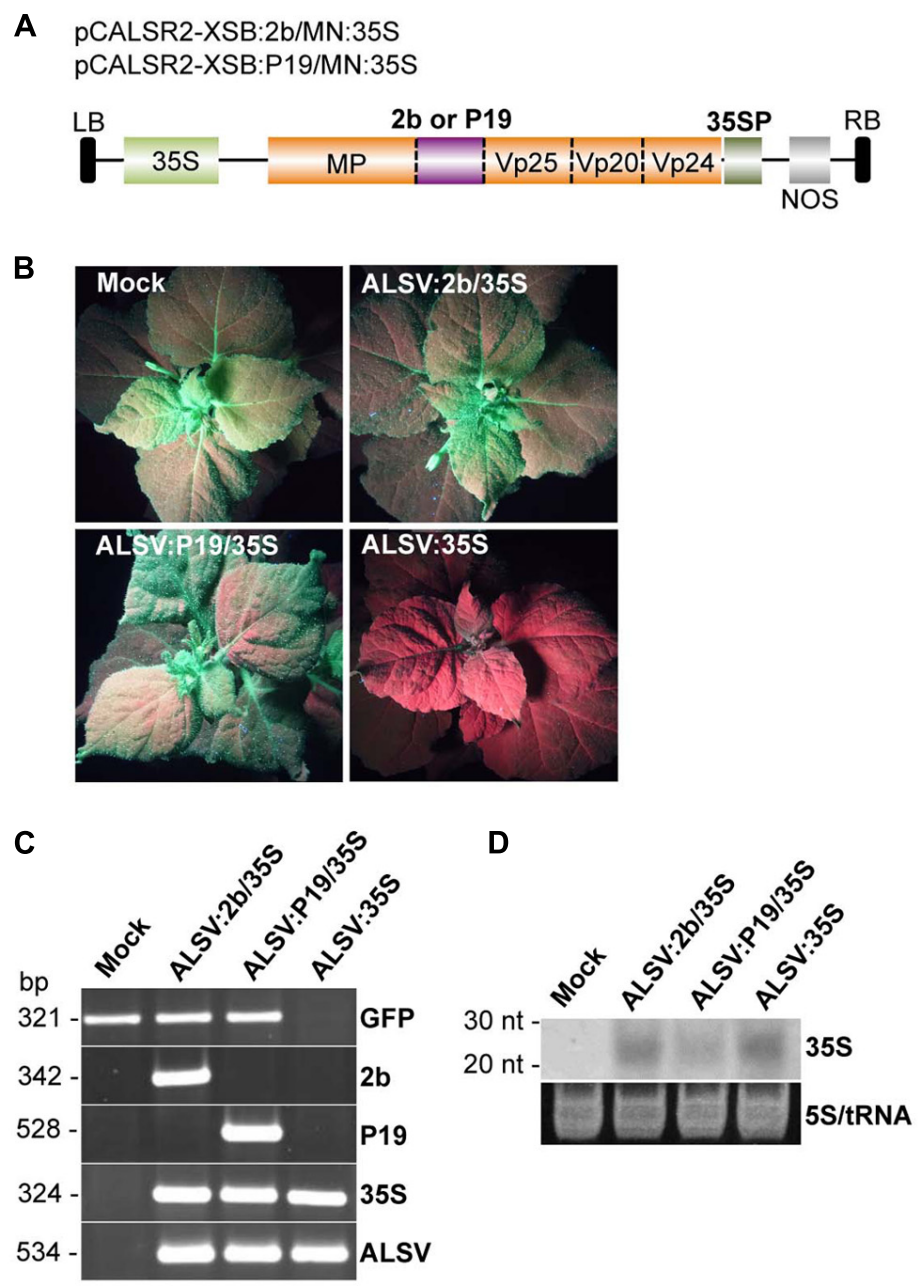
**Induction of TGS by ALSV vectors carrying an RNA silencing suppressor. (A)** Schematic representation of ALSV vectors expressing a plant virus suppressor (2b or P19; pCALSR2-XSB:2b/MN:35S or pCALSR2-XSB:P19/MN:35S). **(B)** *N. benthamiana* line 16c plants expressing GFP and mock-agroinoculated (Mock), agroinoculated with pCALSR1 + pCALSR2-XSB:2b/MN:35S (ALSV:2b/35S), pCALSR1 + pCALSR2-XSB:P19/MN:35S (ALSV:P19/35S), or pCALSR1 + pCALSR2-XSB/MN:35S (ALSV:35S). Plants were photographed under UV illumination with a yellow filter. The red fluorescence reveals that TGS of GFP occurred in ALSV:35S infected tissues. **(C)** Analysis of mRNAs in agroinoculated *N. benthamiana* line 16c plants by RT-PCR. Total plant RNA was extracted from plants agroinoculated with empty vector (Mock), ALSV:2b/35S, ALSV:P19/35S, or ALSV:35S. **(D)** Northern blot analysis of siRNAs corresponding to the CaMV 35S promoter gene. Total RNA was extracted from plants agroinoculated with empty vector (Mock), ALSV:2b/35S, ALSV:P19/35S or ALSV:35S, and hybridized with a sense DIG-labeled RNA probe of the CaMV 35S promoter. The 5S rRNA and tRNA were stained by ethidium bromide and are shown as a loading control.

A recent study showed that CMV-encoded 2b protein promotes efficient epigenetic modification through the transport of the 2b-siRNA complex to the nucleus (Kanazawa et al., 2011). To test whether the 2b protein could more effectively promote ALSV-mediated VITGS, an ALSV vector was constructed to express the 2b protein as well as to silence the CaMV 35S promoter in *N. benthamiana* line 16c plants. The 2b protein was cloned into multiple cloning sites in the coding region of a recombinant ALSV RNA2 vector carrying the CaMV 35S promoter sequence (pCALSR2-XSB/MN:35S) to produce pCALSR2-XSB:2b/MN:35S (ALSV:2b/35S; **Figure 4A**). When the resulting ALSV:2b/35S was used for inoculation of line 16c plants, all plants infected with ALSV:2b/35S retained GFP fluorescence in the upper leaves (**Figure 4B**). A similar result was obtained in line 16c plants infected with ALSV:P19/35S (**Figure 4B**). In contrast, GFP fluorescence disappeared in line 16c plants infected with ALSV:35S (**Figure 4B**). RT-PCR showed that GFP mRNA could be detected from line 16c plants infected with either ALSV:2b/35S or ALSV:P19/35S, but not from line 16c plants infected with ALSV:35S (**Figure 4C**). Northern blotting showed that CaMV 35S-derived siRNAs were detected from the upper leaves of plants infected with ALSV carrying CaMV 35S fragment vectors (**Figure 4D**). These results suggest that viral silencing suppressors (2b and P19) inhibit ALSV-mediated VITGS in plants.

### INDUCTION OF ALSV-MEDIATED VITGS OF AN ENDOGENOUS GENE IN PLANTS

**FIGURE 5:**
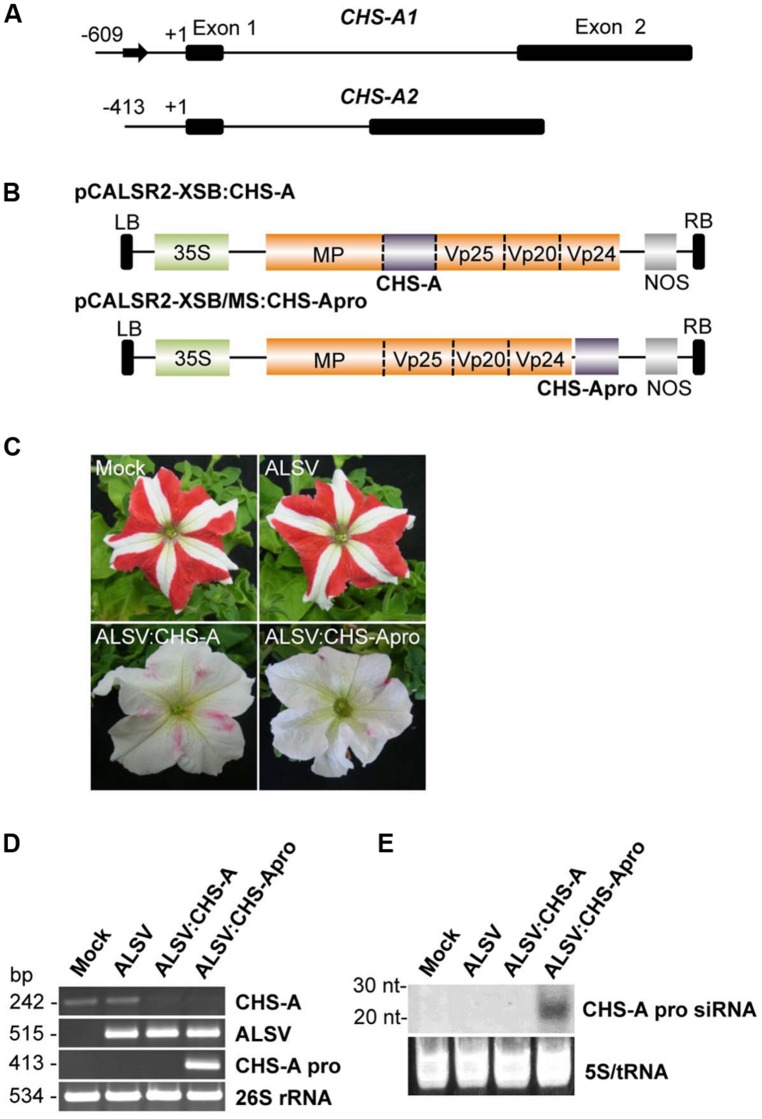
**Induction of TGS by ALSV vector carrying an endogenous petunia gene. (A)** The structure of the *CHS-A* genes. The sequence described by Morita et al. (2012) is in the DDBJ/EMBL/GenBank database under the accession number AB678719. Arrow indicates a transposon-like 179-bp insertion fragment in the *CHS-A1* promoter region. Nucleotide numbering is relative to the transcription start site. **(B)** Schematic representation of ALSV vectors for VIGS (pCALSR2-XSB:CHS-A) and VITGS (pCALSR2-XSB/MS:CHS-Apro) of *CHS-A* genes. **(C)** Petunia flower color phenotypes. Petunia cv. Red Star plants agroinoculated with empty vector (Mock), pCALSR1 + pCALSR2-XSB (ALSV), pCALSR1 + pCALSR2-XSB:CHS-A (ALSV:CHS-A), or pCALSR1 + pCALSR2-XSB/MS:CHS-Apro (ALSV:CHS-Apro). **(D)** Analysis of mRNAs in agroinoculated petunia plants by RT-PCR. Total RNA was extracted from plants agroinoculated with empty vector (Mock), ALSV, ALSV:CHS-A, and ALSV:CHS-Apro. The 26S rRNA was amplified by PCR as an internal control. **(E)** Northern blot analysis of siRNAs corresponding to the *CHS-A* promoter gene. Total RNA was extracted from plants agroinoculated with empty vector (Mock), ALSV, ALSV:CHS-A, or ALSV:CHS-Apro and hybridized with a sense DIG-labeled RNA probe for the *CHS-A* promoter. The 5S rRNA and tRNA were stained by ethidium bromide and are shown as a loading control.

To investigate whether the ALSV-VITGS vector can induce DNA methylation in an endogenous plant gene, the DNA methylation patterns were analyzed in an endogenous gene promoter in petunia plants. The natural bicolor trait of petunia cv. Red Star is caused by post-TGS of the two *CHS-A* gene copies (**Figure 5A**; Morita et al., 2012). The promoter of the *chalcone synthase*-*A* (*CHS-A*) gene, which encodes an anthocyanin biosynthetic enzyme, was cloned into the ALSV RNA2-based vector to produce pCALSR2-XSB/MS:CHS-Apro (**Figure 5B**), and then the recombinant ALSV vector (ALSV:CHS-Apro) was agroinoculated into petunia plants. When petunia plants were infected with wild-type ALSV, the flower color pattern did not change, as also observed with mock-agroinoculated plants (**Figure 5C**). However, petunia plants infected with ALSV:CHS-Apro showed white petal patterns (**Figure 5C**). A similar result was obtained for petunia plants infected with ALSV vector carrying a partial fragment of the *CHS-A* gene coding region (ALSV:CHS-A; **Figures 5B,C**).

Total RNA was extracted from the white tissue of petunia flowers and analyzed by RT-PCR. *CHS-A* mRNA was reduced in plants infected with ALSV:CHS-A and ALSV:CHS-Apro, in contrast to plants treated by mock-agroinoculation or infected with ALSV (**Figure 5D**). The level of *CHS-A* specific siRNA in infected plants was assayed by northern blotting. *CHS-A* promoter specific siRNA was detected from plants infected with ALSV:CHS-Apro, but not from plants inoculated with a mock treatment or with ALSV or ALSV:CHS-A (**Figure 5E**).

**FIGURE 6:**
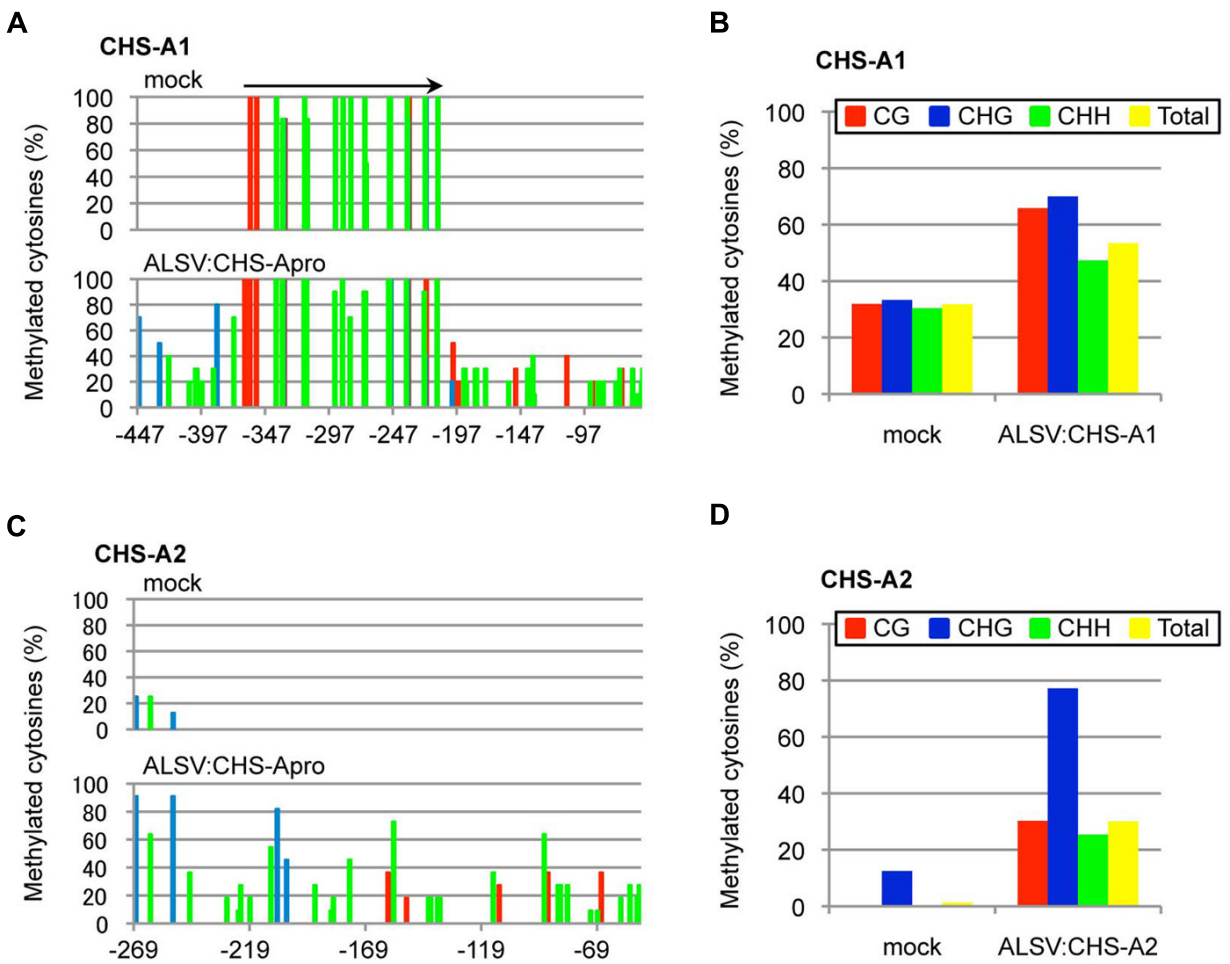
**Cytosine methylation status of the promoter of *CHS-A1* and *CHS-A2*.** Total DNA was extracted from mock-agroinoculated petunia plants and from plants infected with ALSV:CHS-Apro and treated with bisulfite. The red, blue, and green bars respectively represent the percentage of cytosines methylated at the CG, CHG, and CHH sites. Arrow indicates a transposon-like 179-bp insertion fragment in the *CHS-A1* promoter region. **(A)** Cytosine methylation of *CHS-A1.* **(B)** Summary of bisulfite sequencing analysis of the *CHS-A1* promoter gene. **(C)** Cytosine methylation of *CHS-A2.* **(D)** Summary of bisulfite sequencing analysis of the *CHS-A2* promoter gene.

Next, the DNA methylation patterns of the *CHS-A* promoter from white tissue of flowers infected with ALSV:CHS-Apro were analyzed by bisulfite sequencing. Petunia cv. Red Star plants have two copies of the *CHS-A* gene (designated *CHS-A1* and *CHS-A2*; Morita et al., 2012; **Figure 5A**). The *CHS-A* promoter region (nt –447 to –53, corresponding to the transcriptional start site at 1 for the *CHS-A1* promoter and nt –269 to –52 for the *CHS-A2* promoter) has potential transcriptional regulation domains (e.g., an anther box, CACGTG motifs, and TACPyAT boxes; van der Meer et al., 1990). In mock-agroinoculated plants, no cytosine residues at CG, CHG, and CHH sites were methylated except for a 179-bp insertion fragment within the promoter (**Figure 6A**). The *CHS-A1* promoter sequence has a transposon-like insertion fragment (Morita et al., 2012). Bisulfite sequencing showed that the 179-bp insertion fragment region of the *CHS-A1* promoter in mock-agroinoculated plants was highly methylated (**Figure 6A**). The cytosine residues at the CG, CHG and CHH sites including the 179-bp insertion fragment region, were respectively methylated 31.9, 33.3, and 30.4% in mock-agroinoculated plants (**Figure 6B**). In contrast, petunia plants infected with ALSV:CHS-Apro showed methylation at the CG, CHG, and CHG sites including the 179-bp insertion fragment region in 65.8, 70, and 47.3%, respectively (**Figures 6A,B**). The percentage of methylation at the CHH sites in the *CHS-A1* promoter in infected plants was higher than that in the region of the CaMV 35S promoter of line 16C plants (**Figure 3**). This may be due to the presence of transposon-like sequences in *CHS-A1* which were highly methylated in CHH sites in mock-inoculated plants (**Figure 6B**). Cytosine methylation of the *CHS-A2* promoter gene was also found in plants infected with ALSV:CHS-Apro, and the CG, CHG, and CHH sites were methylated 30.3, 77.2, and 25.4%, respectively (**Figures 6C,D**). In mock-agroinoculated plants, a small number of cytosine residues of the *CHS-A2* promoter gene were methylated at CHG (12.5%) and CHH (0.4%) sites (**Figures 6C,D**). These results suggest that transcriptional suppression occurs in plants infected with ALSV vectors carrying a promoter sequence and that the VITGS-derived siRNA induces DNA methylation of the endogenous plant gene.

## DISCUSSION

The VIGS system has been used for functional analysis of genes in diverse plant species. Numerous plant viral vectors including DNA and RNA viruses have been developed (Becker and Lange, 2010). In addition, an advantage of a plant viral-based VIGS system is the ability to introduce viral vectors simply into plants by agroinoculation. Potato virus X (PVX)-based vectors have been developed for gene expression and RNA silencing studies (Baulcombe et al., 1995; Jones et al., 1999), but a PVX-based vector has a limited host range. On the other hand, a tobacco rattle virus (TRV)-based vector has been developed and used for a number of VIGS studies (Liu et al., 2002; Brigneti et al., 2004; Burch-Smith et al., 2004). The advantages of the TRV-based vector are easy induction of RNA silencing via a simple agroinoculation method and large-scale screening for functional gene analysis. The ALSV-based vector constructed in our laboratory has several advantages such as more extensive spreading throughout plant tissue, including growing regions, without any obvious symptoms and the ability to infect economically important crops such as solanaceous plants (tobacco, tomato, potato etc.), legumes (soybean, broad bean, azuki bean, pea, etc.), cucurbits (cucumber, melon, squash, luffa etc.; Igarashi et al., 2009), and fruit trees including apple, pear, peach, plum, citrus, and grapevine (Sasaki et al., 2011, unpublished data). However, the infectivity of ALSV vectors in a pUC-based expression cassette vector (pEALSR1 and pEALSR2L5R5; Li et al., 2004) was not high enough for direct infection of most plants, and the vectors needed to be first inoculated into *C. quinoa* plants by a mechanical inoculation method (infection rate, 0∼50%) for virus propagation, followed by inoculation of virus to the target plants. In this paper we developed an agroinoculation system for the ALSV vector. This system, combined with co-expression of a virus silencing suppressor, increased the efficiency of ALSV-based vector infection in *N. benthamiana* plants.

RNA-mediated TGS could target to a gene promoter in genomic DNA via small RNAs (Pontes et al., 2006; He et al., 2009). A TRV vector has been used for inducing DNA methylation on the promoter region of a transgene (Jones et al., 2001). A CMV vector also effectively induces both transgene and endogenous gene promoter TGS in plant genomic DNA (Kanazawa et al., 2011). These viruses generate dsRNA intermediates, which are targeted by the host defense RNA silencing pathway to produce small RNAs. Therefore, infection of plant viral vectors carrying a promoter gene can induce TGS at homologous regions in the genome. The results in this paper demonstrated that an ALSV-based vector carrying a promoter gene effectively induces TGS on the homologous genomic promoter region. This suggests that ALSV produces virus-derived small RNAs during viral replication, and that the small RNAs themselves may directly move to the nucleus and induce TGS. A recent study has shown that endogenous mobile small RNAs from source cells direct epigenetic modifications of the genome in recipient cells (Molnar et al., 2010).

Cucumber mosaic virus 2b protein binds to small RNAs and the 2b-small RNA complex moves to the nucleus (Kanazawa et al., 2011). Thus, the small RNA-binding activity of the 2b protein is thought to be important for induction of TGS in the nucleus (Kanazawa et al., 2011). In this paper, we showed that viral silencing suppressors (tomato bushy stunt virus p19 and CMV 2b) expressed in an ALSV vector block VITGS in *N. benthamiana* plants (**Figure 4**). Since an ALSV-based vector can effectively induce TGS, a nuclear targeting protein such as 2b may not be required for TGS induction in plants infected with ALSV vectors. At present, we have no idea on this discrepancy between CMV and ALSV systems. As the suppressor activity of the 2b protein increased the infection efficiency of ALSV vectors by agroinoculation (**Table 2**), probably due to the inhibition of RNA silencing induced by ALSV infection, the expression of 2b protein may also reduce the production of small-RNAs that act as inducers of TGS in infected *N. bentamiana.*

The ALSV-based vector system effectively induces DNA methylation in plants via VITGS. In animals, DNA methylation normally occurs at a CG site, whereas the cytosine at CG, CHG, and CHH sites can be methylated in plants. When ALSV:35S was introduced into GFP-expressing transgenic line 16c plants, the targeted CaMV 35S promoter region was highly methylated at CG and CHG sites, and cytosine methylation was also found at the CHH site. In plants, RdDM is required for a dsRNA trigger and *de novo* DNA methylation can be induced by DRM methyltransferases at CG, CHG, and CHH sites (Cokus et al., 2008). In this study, cytosine methylation at CG and CHG sites was highly inherited and resulted in down-regulation of gene transcription in S1 progeny (**Figure 3**). In plants, three methyltransferases (*MET1*, *CMT3*, and *DRM2*) have been identified (Cao et al., 2003; Chan et al., 2005). Two maintenance class methyltransferases, *MET1* and *CMT3*, are required for cytosine methylation at CG and CHG sites, respectively (Henderson and Jacobsen, 2007). In S1 progeny, a high cytosine methylation status (>90%) was found at CG and CHH sites, but not at CHH sites (∼5%) in contrast to CHH sites (∼35%) in S0 plants (**Figure 3**). The cytosine methylation at CG and CHG sites in S1 plants may be maintained by the two maintenance class methyltransferases. Cytosine methylation at CHH sites in S1 plants resulted in *de novo* DNA methylation. This cytosine methylation at CHH sites may be controlled by a *de novo* class methyltransferase (e.g., *DRM2*) or maintained by maintenance class methyltransferases (e.g., *MET1* and *CMT3*; Cao et al., 2003). Previous findings have shown that cytosine methylation at CHH sites at some loci in plants is controlled by *CMT3* and *DRM2* (Cao et al., 2003; Chan et al., 2005). Maintenance of the cytosine methylation at CHH sites in RdDM normally requires for the presence of a dsRNA trigger (Vaucheret, 2006; Zaratiegui et al., 2007). The current results suggest that asymmetric CHH methylation in S0 plants is inherited, and is maintained in S1 plants in the absence of dsRNA, because the recombinant ALSV vector carrying the promoter gene was not detected in S1 plants.

Here, transcriptional silencing of an endogenous gene by its native promoter was demonstrated after recombinant ALSV-based vector infection. The level of DNA methylation of an endogenous gene promoter (e.g., the *CHS-A* promoter) was also induced in wild-type petunia plants infected with ALSV:CHS-Apro (**Figure 6**). A *de novo* class DNA methyltransferase (i.e., *DRM2* and its orthologs) controls the dsRNA-mediated RdDM pathway (Cao et al., 2003; Chan et al., 2005). The methylation status of CG, CHG, and CHH sequences may differ among plant species, or DNA methyltransferase activity may depend on plant developmental stage (Cao and Jacobsen, 2002; Cao et al., 2003). However, no studies on biochemical properties of DRM orthologs from petunia plants have been reported. It will be interesting to see whether the DRM protein expressed by the ALSV vector can increase DNA methylation frequency at target sites in plants.

This study demonstrated that agroinoculation of modified ALSV-based vectors and co-expression of RNA silencing suppressors from diverse plant viruses increased the efficiency of agroinoculation. In addition, the modified ALSV-based vector induced effective TGS of a promoter gene in plants. Thus, the ALSV-based VITGS system provides a useful tool for epigenetic modification and could be used as a technique for biotechnology-based plant breeding (Hartung and Schiemann, 2014).

## Conflict of Interest Statement

The authors declare that the research was conducted in the absence of any commercial or financial relationships that could be construed as a potential conflict of interest.
